# Can People Infer Distance in a 2D Scene Using the Visual Size and Position of an Object?

**DOI:** 10.3390/vision6020025

**Published:** 2022-05-04

**Authors:** John Jong-Jin Kim, Laurence R. Harris

**Affiliations:** 1 Centre for Vision Research, York University, Toronto, ON M3J 1P3, Canada; harris@yorku.ca; 2 Department of Psychology, York University, Toronto, ON M3J 1P3, Canada

**Keywords:** perceived size, perceived distance, pictorial cues, 2D scene perception

## Abstract

Depth information is limited in a 2D scene and for people to perceive the distance of an object, they need to rely on pictorial cues such as perspective, size constancy and elevation in the scene. In this study, we tested whether people could use an object’s size and its position in a 2D image to determine its distance. In a series of online experiments, participants viewed a target representing their smartphone rendered within a 2D scene. They either positioned it in the scene at the distance they thought was correct based on its size or adjusted the target to the correct size based on its position in the scene. In all experiments, the adjusted target size and positions were not consistent with their initially presented positions and sizes and were made larger and moved further away on average. Familiar objects influenced adjusted position from size but not adjusted size from position. These results suggest that in a 2D scene, (1) people cannot use an object’s visual size and position relative to the horizon to infer distance reliably and (2) familiar objects in the scene affect perceived size and distance differently. The differences found demonstrate that size and distance perception processes may be independent.

## 1. Introduction

The relationship between visual size and distance has been obvious to humans for a long time: as an object moves further away, its visual size shrinks in a systematic way. Size constancy, the ability to recognize that an object is the same size despite its visual size changing as its distance from the observer changes, is present from a very young age [1,2], even at birth [3]. It seems likely that the concept is even hardwired in our brains [4,5] as knowing the size and distance of objects is very important to our survival. The primary visual cortex (V1) serves as an important node in the integration of signals from various sources to produce stable perceived size [5]. Chen et al. observed stronger neural responses in V1 to stimuli at far distances compared to stimuli at near distances for objects with the same retinal image size [4], indicating that human V1 can distinguish the distance and size of an object. Such activity is possibly regulated by feedback of distance information from other higher-order areas [6].

Although size constancy may be innate, accurate size estimation requires the development of cognitive ability and the use of strategies such as the distance compensation strategy: deliberately inflating one’s size estimate to compensate for a reduced perceived size at a far distance that supplements size perception [7]. In recent centuries, since the discovery of perspective in art in the 1400s, people have been exposed to images that represent depth on a 2D platform (e.g., paintings, photographs, or monitor screens). Such images use various techniques to simulate natural cues to depth such as texture gradient, perspective, haze, and size constancy. The purpose of this study was to test whether people can determine the egocentric distance of an object in a 2D scene.

### 1.1. Representing Depth in a 2D Scene

In a 3D world, stereopsis is one of the most reliable indications of depth for a person for relatively close objects [8]. The disparities between the views in the two eyes tell the observer how far away an object is from them [9]. The reliability of stereopsis quickly declines with distance as the disparities become too fine to detect although the range of distance over which stereopsis is useful is debatable [10]. Stereopsis is, of course, not at all useful when determining the relative depicted distances of items in a 2D platform as those images of items are presented on a single surface; hence, the simulated distance of different items in such a display must be represented using other depth cues. Here, we investigated the usability of the two depth cues of “visual size” and “distance below the horizon” within a 2D scene.

**Figure 1:**
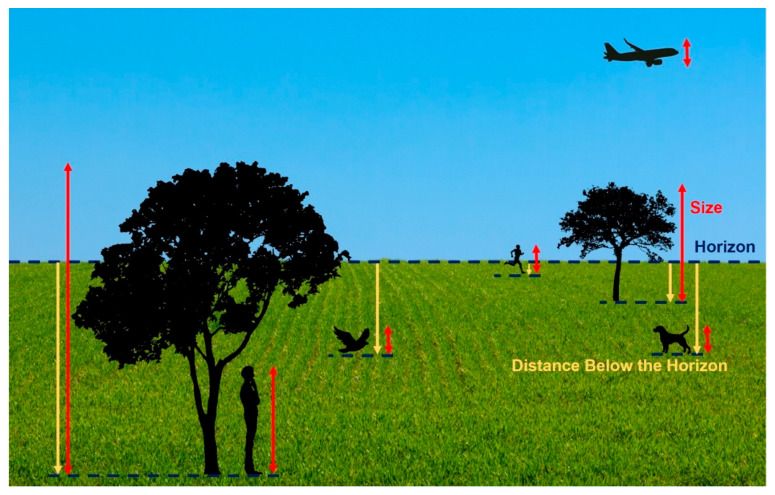
Example of a 2D scene with objects at various distances. Distances are represented with the objects’ (**1**) **size**—objects of smaller visual size (e.g., the running person and the tree on the right side) are perceived as further away compared to the same objects of larger visual size (e.g., the standing person and the tree on the left of the figure); and their (**2**) **distance below the horizon**—objects placed closer to the horizon (the running person and the tree on the right side) are perceived as further away compared to objects further below the horizon (e.g., the standing person and the tree on the left side). The ground plane and the horizon play big roles in depth perception in this example. The running person, the dog, the airplane, and the bird in this scene are all the same visual size. When comparing the person and the dog, it appears obvious the person is further away. However, when comparing them to the bird or the plane, it is ambiguous whether the bird/airplane is: (i) flying at a distance close to the viewer and is smaller in physical size or (ii) flying at a far distance but is larger in size.

As mentioned above, visual size is a useful feature to represent the distance of an object in a 2D scene taking advantage of size constancy. Objects far away are presented smaller in angular size compared to closer objects (see Figure 1). To determine the absolute distance of an object from its visual size, however, the viewer must know the real size of the object. For example, the sizes of the running person, the dog, and the bird in Figure 1 are identical. However, typically, a person is larger than a dog and a dog is larger than a bird. So, it appears that the running person is the furthest from the viewer and the bird is the closest in the scene. If the size of the object were ambiguous—for example, the plane in the scene could be a real airplane and large or a toy airplane and small—then size cannot be used to determine how far away it is.

Another way distances of objects can be represented in a 2D scene is to use the position, i.e., the elevation in the visual field. People can use angular declination below the horizon to determine the absolute distance of an object [11]. In a 2D scene, this can easily be implemented with the objects placed at different elevations. An object placed near the horizon provides a smaller angular declination to the viewer, appearing to be far from them compared to an object placed further below the horizon, appearing to be near (see Figure 1). However, this is only a useful cue for the objects on the ground and not those that are floating or above the horizon. For example, the plane in Figure 1 is the same visual size as the running person and the dog, but it is ambiguous as to whether it is a small plane flying at a close distance or a large plane flying at far distance. Additionally, although the bird in the scene is at the same distance below the horizon and the same visual size as the dog, it is perceived as closer to the viewer than the dog because it is flying (i.e., not on the ground).

### 1.2. Quantifying Depth Cues in a 2D Scene

**Figure 2:**
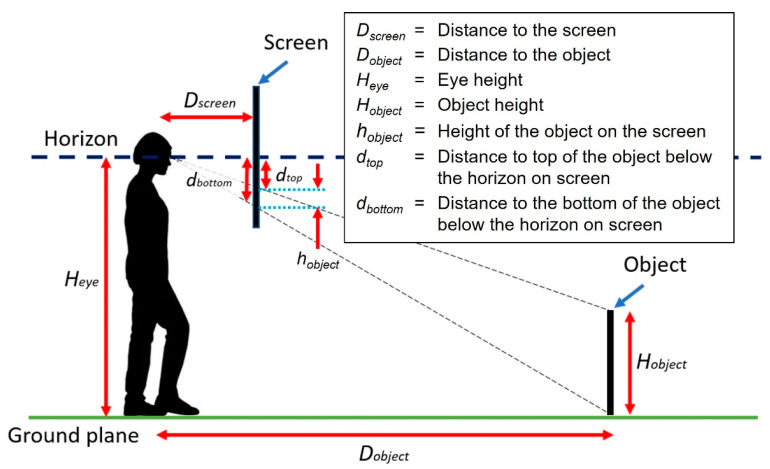
Geometry of an object represented on a 2D platform (the screen). The elevation of the object on the screen can be calculated with: $d_{bottom}=\frac{D_{screen}\times H_{eye}}{D_{object}}$, and the height of the object on the screen can be expressed as *h_object_* or: $\left(d_{bottom}-d_{top}\right)$, where $d_{top}=\frac{D_{screen}\times \left(H_{eye}-H_{object}\right)}{D_{target}}$. Hence, $h_{object}=d_{bottom}-d_{top}=H_{object}\times \frac{D_{screen}}{D_{object}}$.

These depth cues, the elevation and the visual size, representing the distance of an object on a 2D platform are quantifiable using basic geometry. Figure 2 presents an example of a target (with a height of *H_object_*) at a distance (*D_object_*) on the ground represented on a screen placed at a certain distance (*D_screen_*) away from the observer’s eyes (at the height *H_eye_*). The distance of the object below the horizon on screen (*d_bottom_*) and its height on screen (*h_object_*) can be computed using these parameters (see Figure 2). Hence, theoretically, the distance between the observer and the target (*D_target_*) should also be determinable from the object’s position on the screen, i.e., its distance below the horizon (*P*; where *P* = *d_bottom_*):

(1) $$D_{object}=\frac{D_{screen}\times H_{eye}}{P}$$

Based on the dependency of the perceived size of an object on size constancy and Emmert’s law [12], the relationship between the object displayed on a 2D screen and the real object can be represented as (as shown in Figure 2):

(2) $$S=\frac{h_{object}}{H_{object}}=\frac{D_{screen}}{D_{object}}$$

where the size of the object on the screen (*S*) is expressed in proportion to the real target size. Therefore, we can determine the object distance (*D_object_*) from its size on screen in proportion to the true object size (*S*):

(3) $$D_{object}=\frac{D_{screen}}{S}$$

Throughout this paper, the distance below the horizon of the object on screen (i.e., *d_bottom_* from Figure 2 in mm) will be referred to as the ‘position’ of the object (or target) on the screen (*P*) and the ‘size’ of the object (or target) on the screen will be expressed as its size as a proportion of its true physical size (*S*)—see Equation (2).

### 1.3. Hypothesis

In the present study, we evaluated whether people are able to use an object’s position (*P*) and/or its size rendered in a 2D scene (*S*) to estimate an absolute distance. The perceived distance of the target (of a familiar size) was measured indirectly by asking participants to either change a target’s position (*P*) or its size (*S*) to what they perceived to match their smartphone—an object with which they were intimately acquainted—in the scene standing on the ground at a distance (*D_object_*). Perceiving correct distances and sizes requires that the perceived scale of space shrinks with distance [13] and past studies have typically found systematic errors in egocentric distance judgements [8,14]. Past models such as Stevens’ power law (with the exponent 0.67; Table 1) [15] as well as Gilinsky’s formulas for perceived distance (Equation I) [16] both show perceived distance is reduced compared to the real distance. Therefore, it does not seem likely that people can determine the correct absolute distance of an object from a 2D scene which only provides limited cues to distance. Rather, we expect people to underestimate distance compared to the object distance we simulate in the scene in this experiment. However, if people could infer a distance (apparent distance) from position and size using their relationship as shown in Figure 2, then the adjusted positions and sizes of the targets should be consistent.

Here, the participants performed two tasks consecutively. They first adjusted target position while its size was fixed (our Size-to-Position task) or adjusted target size while its position was fixed (our Position-to-Size task). They then adjusted target size while its position was fixed to the size that they had previously set it in the first task or adjusted its position while its size was fixed to the value set in the first task. We hypothesized that people would be able to reliably use a target’s position and size in a 2D scene to infer its apparent distance, and hence be able to adjust target’s size and its position to the values at which it was first presented to them in the first task. To test these hypotheses, we conducted the following experiments.

## 2. Materials and Methods

Three online experiments were conducted following guidelines approved by the York University’s Ethics Review Board and were carried out using the principles of the Declaration of Helsinki. The participants were recruited from the York Undergraduate Research Participant Pool (URPP) and received course credit for their participation. They had to be between 18 and 45 years old to be able to sign up for this study online, but the average age and number of male/females are unknown due to many participants keeping that information hidden from the researcher. All participants gave informed consent before taking part in the experiment.

### 2.1. Apparatus

All experiments were designed and programmed using PsychoPy3 that was run on Pavlovia—an online platform to run experiments [17]. Participants followed a web link provided when they signed up for this study, on their own computer (laptop or desktop). We wanted to simulate an object of a familiar size as the target. Therefore, participants used their personal smartphone as a reference size to compare to visual renderings of their phone shown on the screen. Because the sizes of participants’ screens and their smartphones varied, we needed to know the screen and smartphone sizes for each participant. The participants performed the following tasks at the beginning of the experiment to provide the information to allow us to estimate each participant’s screen and phone size.

#### 2.1.1. Enter Phone Size

**Figure 3:**
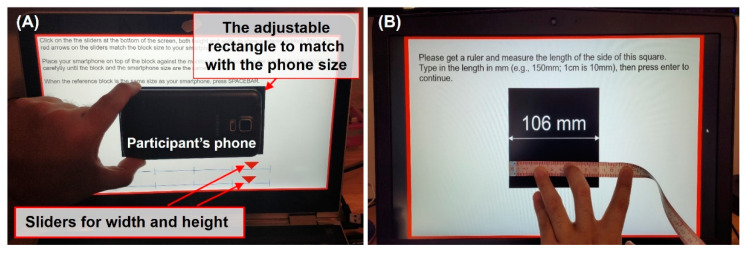
(**A**) Example photo of the phone size setting stage at the start of the experiment. The width (*W_phone_*) and the height (*H_phone_*) of the phones were recorded as a fraction of the height of the screen. (**B**) Example photo of the reference length (*H_reference_*) setting stage.

Participants entered their phone size by physically placing their smartphone on their screen over a black rectangle and then adjusting the rectangle size using sliders—width and height (see Figure 3A). When the rectangle matched their phone size, the participants pressed the spacebar to continue to the next step. The size of the rectangle was saved as the full size of their smartphone. Because the experiment was performed online and participants used their own screens, the screen sizes varied. For this reason, the experiments recorded the phone height and width as a proportion of the height of the screen (using the height units in the PsychoPy3 where the full height of the screen = 1).

#### 2.1.2. Enter Reference Length

Participants measured the length of a black square (each side was ½ of the height of their personal screen) shown on the screen and entered it in mm (see Figure 3B). The participants then pressed the enter key to continue to the next step. This provided us with the actual size of their screen.

#### 2.1.3. Estimating Participant Screen Sizes

Combining the phone size and the reference length entered, we computed each participant’s screen height. We estimated their smartphone sizes by averaging the most popular smartphones in Canada for 2019 [18]—height = 143.3 mm and width 69.4 mm. First the phone size, width (*W_phone_*) and height (*H_phone_*) entered (in proportion to the screen height) were divided by the estimated real phone size and averaged to compute the first estimate of the screen height (H_screen1_):

(4) $$H_{screen1}=\left(\frac{W_{phone}}{69.4}+\frac{H_{phone}}{143.3}\right)\times \frac{1}{2}$$

Because we only asked participants to enter the phone sizes in Exp 1, we used Hscreen1 as the estimated screen sizes for each participant. Then, in Exp 2 and 3, reference length was collected in mm. Since the reference length (*H_reference_*) was ½ of the height of the screen, we multiplied the entered length by 2 to compute the second estimate of the screen height (*H_screen2_*):

(5) $$H_{screen2}=2\times H_{reference}$$

To minimize the disparity between experiments, two estimated screen heights were compared to verify any possible errors and then averaged to compute the final estimate of the screen height for Exp 2 and 3 (*H_screen_*):

(6) $$H_{screen}=\frac{H_{screen1}+H_{screen2}}{2}$$

#### 2.1.4. Visual Stimuli

**Figure 4:**
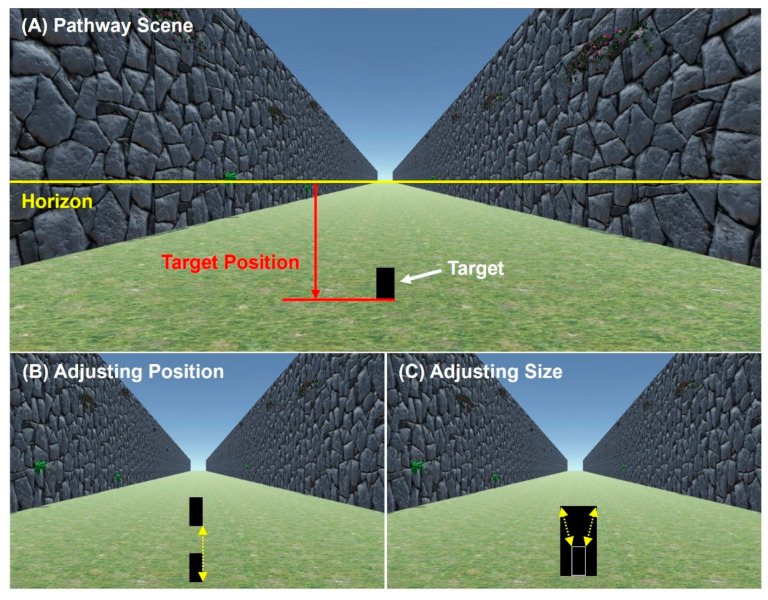
(**A**) The pathway scene with an example of a target (black rectangle) displayed in the scene. The yellow horizontal line is the horizon in the scene—the mid-line of the image. The target position is the distance from the horizon to the target base (red line). Participants were instructed to imagine themselves standing in the pathway looking at their smartphone standing on the ground. (**B**) During the Size-to-Position task, the position of the target could be moved upward by moving the mouse up, and downwards by moving the mouse down. (**C**) During the Position-to-Size task, the target size could be made larger by moving the mouse up, and smaller by moving the mouse down.

The scene displayed was a 2D rendering of a long, grassy path, open to the sky and with stony walls on each side. The scene was created on Unity using a camera view 1.7 m above the ground. The scene contained a grass-textured floor and stone walls on the left and right sides (Figure 4A). The scene was designed to provide limited distance information (i.e., stones on the walls are of unfamiliar shape and size, and there are no other objects in the scene other than the target) so that the observer could focus on the target itself when determining its size/distance. Because the screen sizes and ratios varied between participants, the width and the height of the scene was kept at a 9:5 ratio, where the height of scene rendered on the screen was always the full height of the screen. For most screens, this produced a full screen image.

The target was a black rectangle positioned along the center line of the scene. Participants were asked to imagine that the target was their personal smartphone standing on the ground at some distance from them. Depending on the task (see below), the target could be made smaller/larger or moved up/down in the scene using a mouse.

### 2.2. Tasks

Having calibrated the participant’s equipment with the Enter Reference Length task and the Enter Phone Size task, they then performed two tasks in order: a Size-to-Position task in which they set the position (*P*) of a rendering of their smartphone of fixed size, and a Position-to-Size task in which they set the size (*S*) of their smartphone rendered at a given position. The order of the tasks was varied for each experiment. At the start of the experiment, and throughout the experiment, participants were instructed to keep their eyes 0.5 m from the screen.

#### 2.2.1. The Size-to-Position Task

During the Size-to-Position task, a pathway scene and a target was displayed on the participants’ screen (see Figure 4A). Target size (*S*) was fixed, but participants could adjust its vertical position (*P*). Five different target sizes were used for each test. For each target size, the participants moved the target up or down using their mouse (see Figure 4B) to position it where they perceived it to be at the correct position for that size rendering of their smartphone in the scene. When they thought the target’s size and position were correct, they pressed the spacebar to continue to the next trial.

Target positions were recorded as a fraction of the height of the screen. The full height (bottom to the top of the screen) was defined as 1 unit, where [0,0] represented the center of the screen (see Figure 4A). [0, 0.5] represented the top, and [0, −0.5] represented the bottom of the center line. The horizon extended from [0.9,0] to [−0.9,0] in this notation, hence keeping the 9:5 ratio between the width and the height of the scene.

#### 2.2.2. The Position-to-Size Task

The pathway scene and the target displayed on the participant’s screen were identical to as they were in the Size-to-Position task (Figure 4A). The target position (*P*) was fixed but its size (*S*) could be adjusted. Five different target positions were used for each test. For each target position, participants had to adjust the target size by moving their mouse up and down (Figure 4C). When they perceived the target to be the same size as their smartphone placed at that distance, they pressed the spacebar to end the trial.

The adjusted target sizes were recorded as a fraction of the participant’s actual phone size entered at the Enter Phone Size task, where a value of 1 represented the full size of the phone. For example, a target size of 0.5 corresponds to the target being set to the half the actual size of the phone (in both height and width) rendered according to the geometry of Figure 2.

### 2.3. Methods for Experiment 1

#### 2.3.1. Participants

Out of the 150 people that signed up for the experiment, 122 completed the experiment. After post-test data cleaning (details below), 40 participants had to be removed, leaving 82 participants for the analysis.

#### 2.3.2. Procedure

Experiment 1 started with Enter Phone Size task to obtain the phone size to be used to determine target size throughout. It was followed by the Size-to-Position task, where we measured each participant’s judgement of where each target should be positioned in the scene based on the target size presented. The target size was equivalent to the visual size of their phone (based on the phone size from the Enter Phone Size task) when seen at each of the five distances. The visual sizes were determined using the formula $\frac{D_{screen}}{D_{object}}$, where *D_screen_* is the viewing distance (0.5 m) and *D_object_* is the simulated target distance, 4, 8, 12, 16, and 20 m (see Figure 2).

**Figure 5:**
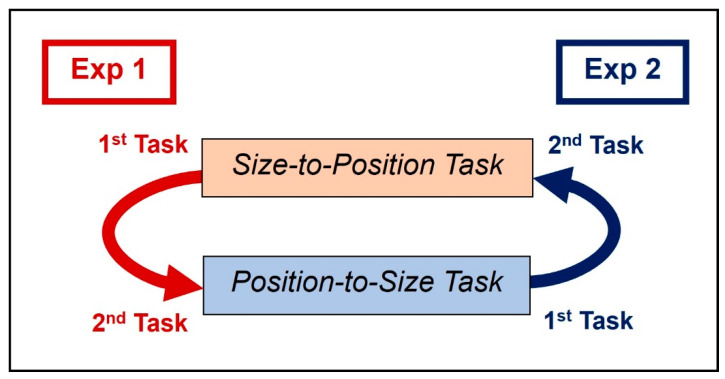
Task orders for Exp 1 and 2. The participants in Exp 1 performed the Position-to-Size task first (1st task) and then the Size-to-Position task (2nd task); and in Exp 2, they performed the Size-to-Position task first (1st task) and then the Position-to-Size task (2nd task). The results from the 1st task (target positions from the Size-to-Position task, and target sizes from the Position-to-Size task) were used in the 2nd task.

After completing the Size-to-Position task, the participant then continued to the Position-to-Size task (see Figure 5 for the task order for Exp 1). We considered the positions where the participant placed targets in the Size-to-Position task to be the position at which they perceived the target as the same size as their smartphone. Therefore, the targets in the Position-to-Size task were presented at the average positions recorded from the Size-to-Position task for each intended target distance.

There was a total of 25 trials (5 trials per target sizes × 5 target sizes) in the 1st task, and 25 trials (5 trials per target position × 5 target positions) in the 2nd task. It took approximately 20 min to complete the experiment.

### 2.4. Methods for Experiment 2

#### 2.4.1. Participants

Out of the 150 people who signed up for the experiment, 100 completed the experiment. After post-test data cleaning (see below), 48 participants had to be removed, leaving 52 participants for the analysis.

#### 2.4.2. Procedure

The test procedures were similar to Exp 1. The Enter Reference Length task (see above) was added following the Enter Phone Size task to obtain additional information about the participant’s screen size. After the Enter Reference Length task, participants performed the Position-to-Size task, where the targets were placed at positions corresponding to the test distances: 4, 8, 12, 16, and 20 m (0.417, 0.191, 0.123, 0.089, and 0.069 below the horizon, where 1 is the full screen height). The positions were determined from the position of the targets when they are placed in the 3D model in Unity which was used to render the 2D scene (see Figure 2 and Visual Stimuli section). Then, the participants continued to the Size-to-Position task, using the average sizes recorded from the Position-to-Size task for each intended target distance (see Figure 5 for the task order for Exp 2).

There was a total of 25 trials (5 trials per target sizes × 5 target sizes) in the 1st task, and 25 trials (5 trials per target position × 5 target positions) in the 2nd task. It took approximately 20 min to complete the experiment.

### 2.5. Methods for Experiment 3

#### 2.5.1. Participants

A total of 210 people completed the experiment (93 in group 1 and 117 in group 2). After post-test data cleaning (see below), 126 participants had to be removed, leaving 84 participants for the analysis (42 in each of two group).

#### 2.5.2. Procedure

**Figure 6:**
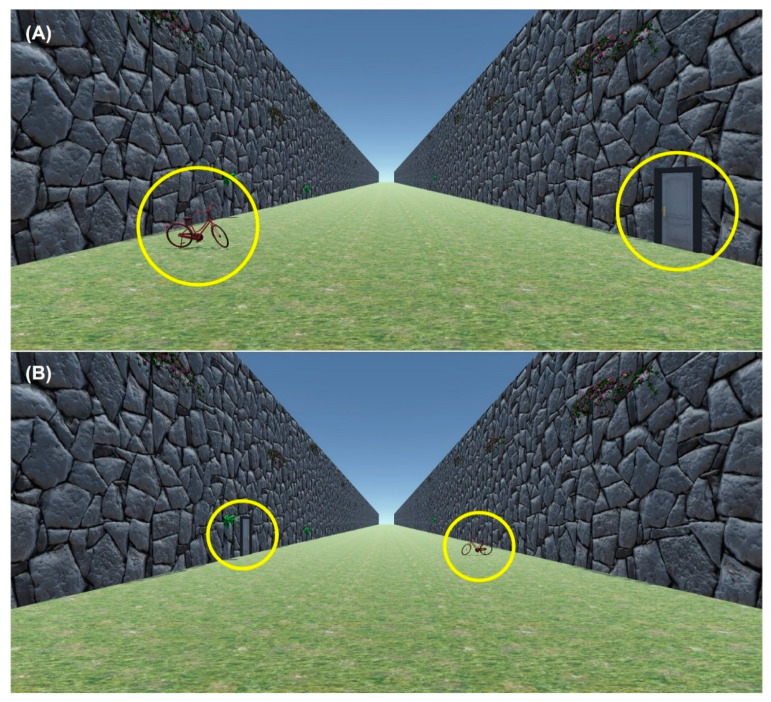
The pathway scene as Figure 4A with familiar objects: a bicycle and a door (highlighted by yellow circles) at near (**A**) and far (**B**) distances. The circles were not present in the display.

The test procedures were similar to Exp 1 and 2 except the 2D pathway scene had familiar objects added to the scene. The familiar objects used were a bicycle and a door. They were either near (at 8 m) or far (at 16 m) from the observer in the rendered scene (see Figure 6). There were 2 conditions: task order and familiar object distance. Groups 1 and 2 were separated by task order: group 1 followed the task order for Exp 2 (see Exp 2 order in Figure 5), and the group 2 followed the task order for Exp 1 (see Exp 1 order in Figure 5).

The order of the familiar object scenes (near or far) was counterbalanced to monitor any order effect of the familiar object position in the scene. In the 1st task, those with odd participant numbers (in both groups 1 and 2) viewed the scene with the familiar objects at the near distance for the first 20 trials then at the far distance for the next 20 trials and the order was reversed for those with even participants numbers. In the 2nd task, 40 trials were presented using the responses obtained from the near trials in the 1st task (20 with near familiar objects—near-to-near; and 20 with far familiar objects in the scene—near-to-far) and 40 trials were presented using the responses from the far trials in the 1st task (20 with near familiar objects—far-to-near; and 20 with far familiar objects in the scene—far-to-far). All 80 trials in the 2nd task were randomly presented to the participants. All participants experienced the four familiar object orders (near-to-near, near-to-far, far-to-near, and far-to-far). Therefore, there were a total of 120 trials for each participant: 40 trials (4 trials per target distances × 5 target distances × 2 familiar object distances) in the 1st task, and 80 trials (4 trials per target distances × 5 target distances × 4 familiar object distance orders) in the 2nd task. It took approximately 30 min to complete the experiment.

### 2.6. Data Analysis

In each experiment, the two tasks were performed in an order which depended on the experiment as described above. The target position or size results from the 1st task were used to set the initial values used in the 2nd task. Therefore, if participants could reliably estimate a target’s visual size from its position and its position from its visual size, then the initial target position or size (presented in the 1st task) should match the final target size or position (results from the 2nd task). To determine whether participants could use target size to determine the correct target position in the scene or use target position to determine the correct target size, we compared the initial target size/position combination to the final target size/position combination after completing the tasks for each experiment. We also compared the target size and position between experiments.

#### Converting Positions to mm Below the Horizon

To convert target positions in proportion to the screen height (*P_screen_*) to millimeters (mm), we multiplied the estimated screen height (*H_screen_*) for each participant (see Section 2.1.3. for details on how that was computed) to the target positions results:

(7) $$P=H_{screen}\times P_{screen}$$

### 2.7. Post-Test Data Cleaning

Each participant’s responses were evaluated for any possible misunderstanding of the tasks and irrational responses due to lack of enthusiasm (e.g., button mashing) that is inevitably found amongst undergraduates running unsupervised experiments online. Participants were removed from analysis if any of the followings were evident:

1. Incomplete dataset;
2. Impossible target position (target position > 0—above horizon);
3. Unfeasible screen/phone sizes (i.e., the phone height-to-width ratio > 0.7 or <0.3, and the discrepancy in estimated screen heights from the phone size and the reference size is larger than 50 mm);
4. Uncorrelated responses to the target size/position conditions (suspected of not paying attention to the task, i.e., standard deviation of their responses was 0 in one or more conditions).

## 3. Results

### 3.1. Experiment 1: The Position-to-Size Task and then the Size-to-Position Task

In Exp 1, participants first adjusted the position of targets that were set to the sizes they should be when viewed at 4, 8, 12, 16, or 20 m (the Size-to-Position task) so that they appeared correct. Then, in a second task, they adjusted the size of targets that were positioned at the locations they had set in the 1st task (the Position-to-Size task). We conducted a repeated-measures, i.e., within-subject, analysis of variance (ANOVA) to analyze the differences among the mean sizes (task order: initial vs. final sizes) between the simulated target distances (see Appendix A: Table A1 for the results). The main effect of task order was significant, F(1, 81) = 33.034, *p* < 0.001, η^2^ = 0.207. The main effect of simulated target distance was also significant, F(4, 324) = 130.388, *p* < 0.001, η^2^ = 0.124. These main effects were qualified by a significant interaction between the task order and simulated target distance, F(4, 324) = 6.958, *p* < 0.001, η^2^ = 0.007. Further evaluations with pairwise t-test using Bonferroni correction, adjusting the alpha level to accommodate multiple comparisons and maintain the familywise error rate of 0.05, showed that the final sizes were significantly larger than the initial sizes for each target distance (*p* < 0.001), which suggests that the participants could not successfully adjust targets to their original sizes from their positions in the 2nd task, even though they had themselves placed the targets there in the 1st task. It was also revealed that the final sizes for the targets simulated at closer distances were significantly larger than those simulated at further distance (*p* < 0.001) except for those between 12 and 16 m (*p* = 1.000) and between 16 and 20 m (*p* = 1.000). These results suggest that participants could match the target sizes in the correct order based on their simulated distances up to 12 m (See Table 1A for the average values from Exp 1).

We also conducted a single-factor repeated-measures ANOVA to compare the positions at which targets were placed in the Size-to-Position task (1st task) with the simulated target distances (see Appendix A: Table A2 for the results). A significant main effect of position was found, F(4, 324) = 143.443, *p* < 0.001, η^2^ = 0.639, which was followed by pairwise t-test using Bonferroni correction. The positions were significantly different for each target size (*p* ≤ 0.05) except for those between 16 and 20 m (*p* = 1.000). That is, participants could match target size and position in the correct order in the scene up to 16 m away for their positions and up to 12 m away for their sizes. Participants may not have been able to infer target distances from their sizes or positions in a 2D scene because they could not match the targets’ final sizes to their initial sizes. 

**Figure 7:**
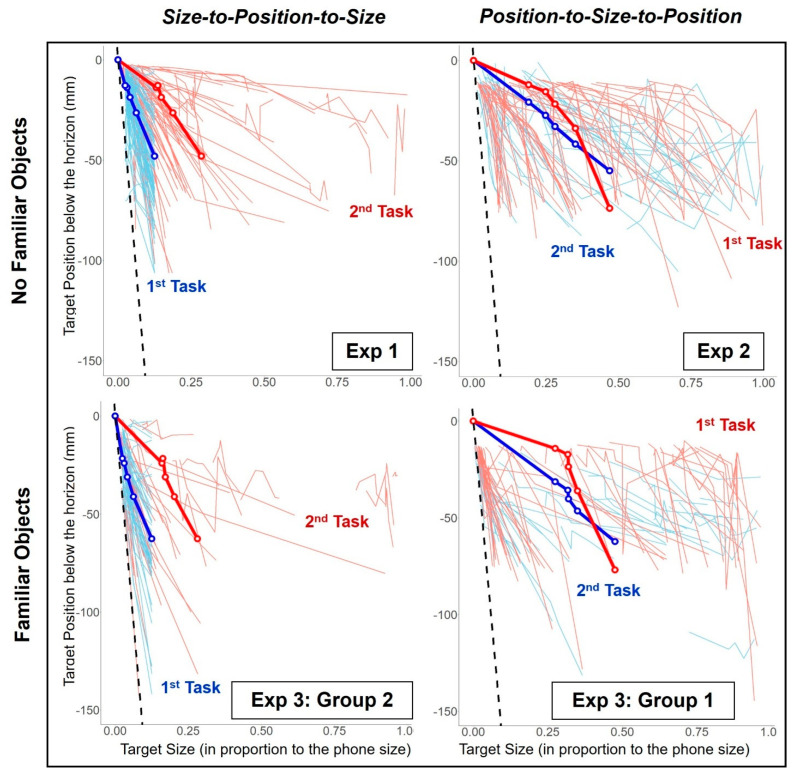
Participants’ settings for all tasks. Target position plotted as a function of target size. Top row, with no familiar objects (**Exp 1** and **Exp 2**); bottom row, with familiar objects (**Exp 3**). Left column, the Size-to-Position task first followed by the Position-to-Size task; right column, the Position-to-Size task first followed by the Size-to-Position task. The dashed black lines represent target sizes and positions with the correct geometry. The solid light-colored lines are the average responses from each individual participant and the darker-colored lines with white dots represent the mean of responses from all participants for each task, where the dots are the means for each simulated target: the Size-to-Position task (**Blue**) and the Position-to-Size task (**Red**). (The means and the standard errors can be found in Table 1).

Individual participant responses are shown in Figure 7 (Exp 1), which plots the position set as a function of size for the two tasks. The data patterns for the two tasks are very different from each other and from the black dashed line which indicates the correct geometry. To discover whether the order of the tasks mattered, we conducted Experiment 2 in which the task order was reversed.

### 3.2. Experiment 2: Size-to-Position then Position-to-Size

In Exp 2, the task order was the reverse of that used in Exp 1: participants first adjusted target sizes from their given positions simulated to be at 4, 8, 12, 16, and 20 m (the Position-to-Size task). Then in the 2nd task, they adjusted the target positions for targets set to the sizes that they had just set for each simulated distance in the 1st task (the Size-to-Position task). The data are plotted in Figure 7 (Exp 2).

A repeated-measures ANOVA was conducted to compare the positions (task order: initial vs. final positions) between the simulated targets distances (see Appendix A: Table A3 for the results). The analysis revealed a significant main effect of task order, F(1, 51) = 6.582, *p* = 0.013, η^2^ = 0.010, and a significant main effect of simulated target distance, F(4, 204) = 385.818, *p* < 0.001, η^2^ = 0.664. These main effects were qualified by a significant interaction between the task order and simulated target distance, F(4, 204) = 46.905, *p* < 0.001, η^2^ = 0.077. Further evaluations with pairwise t-test using Bonferroni correction showed that the final positions were significantly different compared to the initial positions for each target distance (*p* < 0.050), which suggests that the participants could not successfully adjust targets to their original positions from their sizes in the 2nd task which they had set in the 1st task. It was also revealed that the final positions were significantly further below the horizon for targets that were simulated at closer distances than those simulated at further distances except for the targets between 12 and 16 m (*p* = 0.185). These results suggest that participants could place the targets in the correct order based on their simulated distances up to 12 m (See Table 1B for the average values from Exp 2).

In addition, a single-factor repeated-measures ANOVA was conducted to compare the sizes set in the Position-to-Size task (1st task) between intended target distances (see Appendix A: Table A4 for the results). A significant main effect of task was found, F(4, 204) = 68.352, *p* < 0.001, η^2^ = 0.573. Pairwise t-test using Bonferroni correction showed that the positions were significantly different for each target size except for those between 12 and 16 m (*p* = 0.770). Therefore, participants could adjust the target sizes to the correct order in the scene up to 12 m.

These results are similar to the findings from Exp 1. The results further suggest that participants could adjust the target sizes and positions to the geometrically correct order to a degree, but that their responses were again very different from the correct geometry (see Figure 7—Exp 2; the blue and the red lines compared to the black dashed line). From the results of Exp 1 and 2, it appears that the participants were not able to infer distances from their sizes or positions in a 2D scene.

### 3.3. Experiment 3: Familiar Objects in the 2D Scene

In Exp 3, we repeated Exp 1 and 2 with the simulated target distances at 4, 8, 12, 16, and 20 m but provided an additional distance cue—familiar objects (a bicycle and a door, see Figure 6) in the scene. Group 1 followed the task order of Exp 2 and Group 2 followed the task order of Exp 1. The familiar objects in the scene were either at near or far distances, the order of which was counterbalanced during the experiment (see Section 2.5.2 for details).

We conducted a repeated-measures ANOVA comparing target positions for Group 1 and sizes for Group 2 to determine whether there was any effect of the order of the familiar object positions: near-to-near, near-to-far, far-to-near, and far-to-far. The analysis revealed no significant differences between familiar object orders for Group 1, F(3, 123) = 0.117, *p* = 0.950, η^2^ < 0.001, or Group 2, F(3, 123) = 0.173, *p* = 0.915, η^2^ < 0.001. Therefore, we pooled the participant responses by averaging the sizes and positions across the familiar object order conditions for the following analyses.

#### 3.3.1. Group 1: Position-to-Size-to-Position

We performed a repeated-measures ANOVA to compare the positions (task order: initial vs. final positions) between the simulated target distances (see Appendix A: Table A5 for the results). The analysis revealed a significant main effect of task order, F(1, 41) = 29.855, *p* < 0.001, η^2^ = 0.054, and a significant main effect of simulated target distance, F(4, 164) = 422.301, *p* < 0.001, η^2^ = 0.658. We also found a significant interaction between task and target position, F(4, 164) = 61.639, *p* < 0.001, η^2^ = 0.090. Pairwise comparisons using Bonferroni correction showed that the final positions were significantly different from the initial positions for each simulated target distance (*p* ≤ 0.001). Further evaluation also showed that the final positions were significantly further below the horizon for the targets simulated at closer distances than those simulated at further distances except for those between 12 and 16 m (*p* = 0.574), and between 16 and 20 m (*p* = 0.639). These results are consistent with the previous results from Exp 2 (see Table 1B for the average values from Exp 3: Group 1).

A single-factor repeated-measures ANOVA was also conducted to compare the sizes matched in the Position-to-Size task (1st task) between simulated target distances—see Appendix A: Table A6 for the results—and a significant main effect of task was found, F(4, 164) = 40.487, *p* < 0.001, η^2^ = 0.497. Pairwise t-test using Bonferroni correction showed that the sizes were significantly different for each intended target distance except for those between 12 and 16 m (*p* = 0.099) and 16 and 20 m (*p* = 1.000).

#### 3.3.2. Group 2: Size-to-Position-to-Size

We performed a repeated-measures ANOVA to compare the sizes (task order: initial vs. final positions) between the simulated target distances (see Appendix A: Table A7 for the results). The analysis revealed a significant main effect of task order, F(1, 41) = 12.123, *p* = 0.001, η^2^ = 0.193, where the final target sizes were significantly larger compared to the initial sizes on average (see initial and final sizes for Exp 3: Group 2 in Table 1A). In addition, there was a significant main effect of simulated target distance, F(4, 164) = 64.329, *p* < 0.001, η^2^ = 0.067. However, there was no significant interaction between order and intended target distance found, F(4, 164) = 0.916, *p* = 0.456, η^2^ < 0.001.

In a single-factor repeated-measures ANOVA conducted to compare the positions set in the Size-to-Position task (1st task) between simulated target distances (see Appendix A: Table A8 for the results), a significant main effect of simulated target distance was found, F(4, 164) = 77.179, *p* < 0.001, η^2^ = 0.653. Pairwise t-test using Bonferroni correction showed that the positions were significantly different for each target size except for those between 12 and 16 m (*p* = 0.099) and 16 and 20 m (*p* = 1.000). These results are similar to those found in Exp 1.

### 3.4. Between Experiments Analysis

#### 3.4.1. Effect of Familiar Objects on the 1st Task

When comparing the adjusted target sizes from the Position-to-Size task in Exp 2 and Exp 3—Group 1, responding to the initial target positions, we used a repeated-measures ANOVA. No significant effect of familiar object was found, F(1, 92) = 0.498, *p* = 0.482, η^2^ = 0.005. However, when comparing the positions set during the Size-to-Position task in Exp 1 and Exp 3—Group 2, responding to the initial target sizes, there was a significant effect of familiar object, F(1, 122) = 20.267, *p* < 0.001, η^2^ = 0.065. These results suggest that familiar objects in a 2D scene did not influence the participants’ judgement of the target size based on target position (Position-to-Size), but they affected their judgement of the target position based on target size (Size-to-Position).

#### 3.4.2. Effect of Familiar Objects on the 2nd Task

When comparing the target positions set during the Size-to-Position task in Exp 2 and Exp 3—Group 1, responding to the target sizes they adjusted to in the 1st task, we used a repeated-measures ANOVA. A significant effect of familiar object was found, F(1, 92) = 6.628, *p* = 0.012, η^2^ = 0.032. However, when comparing the adjusted target sizes from the Position-to-Size task in Exp 1 and Exp 3—Group 2, responding to the target positions they set in the 1st task, no significant effect of familiar object was found, F(1, 122) = 0.188, *p* = 0.665, η^2^ = 0.001. These results are consistent with the findings from the comparisons of the 1st task, where familiar objects in a 2D scene did not influence the participants’ judgement of the target size based on target position (Position-to-Size), but they affected their judgement of the target position based on target size (Size-to-Position).

## 4. Discussion

In our study, participants positioned a rendition of their cell phone of a given size to where they thought it should be in a 2D scene, the Size-to-Position task, or they adjusted its rendered size until it appeared correct while it was at some fixed position in the scene, the Position-to-Size task. The distance of a given object determines its image size. Hence, participants would have had to estimate the distance to the rendition of their cell phone to be able to adjust its size correctly and vice versa. Given the known familiar physical size of their phone, we hypothesized that they should have been able to adjust the target’s position below the horizon and the size at which it should appear. Instead, in all the experiments, the final target size/position combinations did not match the initially simulated values.

We interpret our results as indicating that our participants could not infer target position from its size or its size from its position, which implies that our participants were not able to estimate the distance to the target reliably. In general, they made the rendition too large or placed it too close to the horizon (too far away) compared to the geometrically correct values (see Figure 7). Pictorial cues such as wall patterns (although the stone sizes for our walls were ambiguous compared to standard sized bricks) and linear perspective from the intersections between the walls and the floor can help in people’s distance perception [19] but participants could not use these cues to determine absolute distance. This may be due to the 2D scene being seen as smaller (minified) as was found in past studies when a real-world scene that was presented on a synchronized image display had to be magnified substantially to be seen as correct [20]. If the 2D space were perceived as smaller than simulated, then an object would have had to be made larger for it to appear to be the correct size and placed further away for it to appear to be at the correct distance, which is what our participants did on average. Our results could not be represented using Stevens’s power law [15] or Gilinsky’s formulas for perceived size and distance [16]. Both models propose underestimation of an object distance, whereas our results show the opposite effect for some tasks (i.e., the Size-to-Position task). Additionally, the responses from each individual participant varied greatly, therefore making it difficult to be represented using a single model.

Overall, it would seem that our participants could not use a familiar object’s position (angular declination below the horizon) or its visual size to derive its absolute distance in a 2D scene. Participants not being able to infer absolute distance from size is consistent with past studies of perceived size and distance in the real world [21]. However, not being able to infer distance from target position is not consistent with previous research—where angular declination below horizon helped in distance perception [11]—which may be due to using a 2D scene. Ooi et al. demonstrated that the perceived eye level is important in computing the angular declination below horizon [11]. In the 2D scene presented in our experiment, the ground information from their feet to the bottom of the screen was missing and participants may therefore have misperceived the eye level which was set to a fixed value of 1.7 m. Dixon et al. suggested that eye level in general is not scaled correctly when people view non-immersive displays because the altitude of the horizon is indeterminate [22]. Misperceived eye level would then lead to misperceived angular declination of the target, resulting in errors when determining a target’s distance from its position. Although participants could not determine the absolute distance of the targets from their size and position in the scene, they could use what they saw to, at least mostly, determine the correct distance order. In the real world, a given object at a further distance from the observer has a smaller visual angle compared to that same object viewed at a closer distance. When viewed more distantly, the object will also be closer to the horizon in the visual field. In all experiments, our participants set targets that were closer to the horizon to smaller sizes compared to the targets further below the horizon in the Position-to-Size tasks. They also placed the smaller targets closer to the horizon compared to the larger targets in the Size-to-Position tasks. This shows that they had some idea of the three-dimensional nature of the world that was depicted. Gogel et al. found that the perceived absolute size and distance of an object were positively correlated, but that the ratio between them varied rather than following strict size–distance constancy rules [21]. Our results are consistent with this idea. When the horizon is clearly defined in a picture, observers can use distance to the horizon to determine relative distance to objects and ignore other possibly erroneous information such as the height in the picture plane, i.e., “the distance from the picture’s lower border to the bottom of the object” [23] (p. 445). It would seem that our participants could not derive absolute distances from target size or position, but they could correctly infer relative distances using the angular declination below the horizon and size constancy. Most past studies on perceived size and distance have been conducted in the real or virtual environment in a 3D space (e.g., [4,17,21,24]). When using a 2D image, they were designed to provide sufficient distance information, such as using a live video or a photo of a real scene (e.g., [13,20]), rather than to limit the distance cues as we did in this study. Our study fills this methodological gap in the literature and show that people can judge relative distance to the objects in a 2D scene from using only their visual sizes or positions.

Out of the five targets rendered at distances between 4 and 20 m, participants could do this for targets at 4–12 m (corresponding to the base of the object 76.9 to 21.7 mm below the horizon on average; see Table 1). The horizon ratio, the ratio between the visual height and the distance below the horizon of an object in pictures, can help judge relative sizes which are typically most accurate at eye level, i.e., near horizon [25,26]. Instead, our participants were less accurate in determining the relative sizes of targets closer to the horizon (simulated distance beyond 12 m). This may be due to the difference in the size of the target used. Bertamini et al. used poles at 60% of the observer eye height (the shortest pole was 96 cm) [26], whereas our participants used their own smartphone as reference size (approximately 14.3 cm). As objects are simulated at further distances, their angular size and displacement from the horizon become smaller, hence more difficult to distinguish as the differences in size and distance also become smaller. The largest difference between the targets simulated beyond 12 m were 7 mm for positions and 0.03 for sizes (approximately 4.3 mm for height) which correspond to visual angles of 0.8° and 0.5°, respectively. These differences may have been too small for the participants to distinguish them.

### 4.1. Why Is There a Task Order Effect?

How well our participants performed on the tasks we set them depended, unexpectedly, on the order in which they performed them. The targets positioned based on their size, the Size-to-Position task, were more geometrically accurate compared to the target sizes chosen for a given position, the Position-to-Size task, when they performed the Size-to-Position task first (i.e., blue lines were closer to the dashed line compared to the red lines in Figure 7 Exp 1 and Exp 3: Group 2). However, when they performed the Position-to-Size task first, the size was consistently set too large and subsequently performed the Size-to-Position task did not improve their accuracy. In the 1st task, participants were presented with targets that were set to the geometrically correct size (for the Size-to-Position task) or position (for the Position-to-Size task). Based on these results, it appears that participants were more accurate at placing targets at the geometrically correct positions when the correct target sizes are presented to them during the Size-to-Position task. However, being presented with targets at the correct position during the Position-to-Size task did not help them match the targets to the correct sizes. Our data confirm that the absolute distance of an object may not be determined from its size and position presented in a 2D scene, but it is unclear why participants’ responses differed between the tasks. Such an order effect suggests there might be fundamental differences between these tasks. Do people perceive size and distance differently depending on the task they are performing?

Despite the popularity of the size–distance hypothesis, studies have shown that size perception and distance perception may, to some extent, be independent from each other [27,28]. Kim suggested “size and distances are two independent perceptual processes with each determined directly by the corresponding information sources” [28] (p. 16). Haber and Levin (2001) also claimed that size perception is based on properties of the object such as prior knowledge or experience that the observer had, and that distance perception is based on the environmental information that describe its distance. The two tasks used in the present study asked the participants to determine different aspects of the targets. In the Size-to-Position task, they had to determine target position (i.e., their distances), while in the Position-to-Size task, they had to determine target size. If size and distance perceptions are fundamentally different, then this might explain the differences between the two tasks shown here. That is, being presented with geometrically correct target sizes results in more accurate estimates of target positions in the Size-to-Position task but being presented with correct positions did not result in more accurate target sizes in the Position-to-Size task.

During the Size-to-Position task, they may have used the environment sources (the 2D scene) to determine the target position and focused on placing each target based on where they saw the targets to be in the environment (the pathway scene), ignoring the visual sizes of the targets. If this were the case, then it is possible that participants simply distributed targets along the pathway in the scene based exclusively on their relative sizes. The targets were still placed in the appropriate order, which shows they understood the overall relationship between size and position in a 2D scene. The plots for individual participants shown in Figure 7 show large individual differences. Some participants set target sizes that were so large that their correct geometrical position would have been too close to even be rendered on the screen during the Size-to-Position task (Figure 7, refer to the individuals on the far-right side of the plots). However, instead of placing targets clustered towards the bottom of the screen, they still placed targets distributed along the pathway, further suggesting that they could not use the visual size of the targets to determine their correct positions in a 2D scene.

Similarly, during the Position-to-Size task, many targets were made much larger than their geometrically correct size, despite their position in the scene. The participants may have been unable to use the environment (2D pathway scene) and focused instead on matching the targets to the physical size of their smartphone. When viewing a picture, there are two distinct distances a person can perceive simultaneously: (1) a distance from the eye to the picture, and (2) the distance from the point of view of the picture, i.e., within the three-dimensional scene in the picture [29]. These participants may have failed to perceive the scene as a space within the picture during this task. Some participants, however, were able to use the size and position of targets to determine their geometrically correct distances (Figure 7; refer to the individuals on the far-left side of the plots, close to the geometrically correct lines). Individual differences have been shown in past experiments evaluating absolute sizes and distances for objects beyond 3 feet (e.g., [30,31]); Higashiyama suggested that there may be different populations of observers using different types of strategies [31]. Our participants seem to have used different individual strategies when determining the size of an object as opposed to when determining its distance. These results demonstrate the independence of the size and distance perception further, at least in a 2D scene.

### 4.2. Can Familiar Objects Improve Object Distance Judgements in a 2D Scene?

There are many studies looking at the effect of familiar size on determining the perceived distance of a familiar object (e.g., [27,32,33]). However, results are mixed as to how familiarity affects perception. Hochberg and Hochberg suggested that familiar size may not affect our perception of depth at all [32]. Changing the visual size of a familiar object can sometimes affect its perceived distance [33], but this may depend on the person’s viewing attitude, i.e., using different strategies. Being instructed to use the knowledge the person has about an object can result in them relying less on the perceptual information [34,35], cognitively fixing the absolute size of an object in their mind and only adjusting its apparent distance according to its visual size as Fitzpatrick et al. showed in their study [33]. Haber and Levin found that an observer’s familiarity with an object helped determine their perception of the size and distance of a far object (50–100 m) where distance information was limited, but not for close objects (0–50 m) where distance information was clear [27]. They also found that people were more accurate at determining an object’s size and distance when a given familiar object’s size varied less in general, e.g., bikes vary less in size compared to house plants [27].

In this study, the targets represented participants’ smartphones which they are very familiar with, but these familiar visual stimuli were displayed in a 2D scene which provided limited distance information. Recognizable familiar objects in the environment can help in determining the size and distance of other objects [36]. Therefore, adding a bike and a door to the scene—two of the items used by Haber and Levin as objects with low variance in size (see [27] Table 1, p. 1142)—was expected to improve participants’ size and distance perception. Our results show familiar objects in the scene did indeed influence participants’ responses in the Size-to-Position task. Having familiar objects in the scene resulted in participants’ responses being more geometrically correct in the Size-to-Position task. It appears that familiar objects may play a role as anchors which observers can use when determining an object’s position from its size.

The familiar objects, however, did not affect responses in the Position-to-Size task. Interestingly, therefore, the objects seem to have helped determine a target’s position but not its size in a 2D scene. This does not align with the past findings, where familiar size was found to affect both perceived size and distance [24]. Maltz et al. found people perceived Rubik’s cubes as larger and further away than dice when they were matched to the same physical sizes and distances [24]. However, the familiar size affected size perception more when viewing an object monocularly compared to when viewing it binocularly. Additionally, judgement of size was found to vary with depth information in the real-world scene displayed on a screen, but judgement of distance did not [37]. Our data suggest that when viewing an object in a 2D scene, the presence of familiar objects does not affect perceived size and distance equally, which is a novel finding that, as far as we know, has not been observed before. The differences in the effect of familiar size on our tasks further demonstrates that size and distance perception processes are independent.

### 4.3. Limitations and Future Studies

The present study was conducted online, with each participant using their own computer screen which provided a limited field of view, especially in the vertical dimension. The 2D scene was presented on a regular screen and much of the ground information was missing. Unlike in the lab, we did not have the ability to blank out the rest of the world so that only the screen was visible or to fix our participant’s eye height above the simulated ground plane. Accurate distance judgements require a person’s visual system to form a ground-surface representation which uses near-ground-surface information as its foundation [38]. The lack of sufficient ground plane information in our displays may have contributed to our participants not being able to infer correct distances from target sizes and positions. Future studies should utilize a larger screen, fully extended to the floor, which may allow more accurate distance perception even when viewing an object on a 2D screen.

Although we instructed participants to imagine themselves standing in the pathway scene, some may not have been able to do so effectively. If they failed to consider the environment in the 2D scene as a life-size pathway but instead viewed it like a painting, then they would not have perceived it at the correct scale. The perceived size of an object is “driven from the underlying scale of the environment…within which individual objects are located” [39] (p. 15). Misperceiving the scale of the environment would result in misperceiving the size of an object, leading to inaccurate perceived distance. More immersive experience of the scene, such as in a cave automatic virtual environment (CAVE) or other virtual environment, would help observers perceive an environment at the correct scale, hence perceiving object size correctly and perhaps leading to a more accurate determination of its distance.

## Figures and Tables

**Table 1:** Average Initial and Final Sizes/Positions for All Tasks.

**A.** **The Average Initial and Final Target Sizes for Size-to-Position-to-Size Task Order**					
	**4 m**	**8 m**	**12 m**	**16 m**	**20 m**
Initial size	0.125	0.063	0.042	0.031	0.025
Position (Exp 1) * (mm)	−48.0 ± 2.8	−26.5 ± 1.8	−18.7 ± 1.4	−13.9 ± 1.3	−12.8 ± 1.3
Position (Exp3: Group 2) * (mm)	−62.6 ± 4.9	−41.2 ± 3.0	−31.1 ± 2.1	−24.1 ± 1.8	−21.6 ± 1.8
Final size (Exp 1) **	0.29 ± 0.02	0.19 ± 0.02	0.15 ± 0.02	0.13 ± 0.02	0.14 ± 0.03
Final size (Exp 3: Group 2) **	0.28 ± 0.04	0.20 ± 0.04	0.17 ± 0.04	0.16 ± 0.04	0.16 ± 0.04
**B.** **The Average Initial and Final Target Positions for Position-to-Size-to-Position Task Order**					
	**4 m**	**8 m**	**12 m**	**16 m**	**20 m**
Initial position (Exp 2) (mm)	−73.7 ± 1.5	−33.8 ± 0.7	−21.7 ± 0.4	−15.6 ± 0.3	−12.1 ± 0.2
Size (Exp 2) **	0.47 ± 0.04	0.35 ± 0.03	0.28 ± 0.03	0.25 ± 0.03	0.19 ± 0.03
Final position (Exp 2)* (mm)	−55.0 ± 2.6	−41.7 ± 2.3	−33.0 ± 2.4	−27.4 ± 2.0	−20.9 ± 2.2
	**4 m**	**8 m**	**12 m**	**16 m**	**20 m**
Initial position (Exp 3: Group 1) (mm)	−76.9 ± 2.7	−36.1 ± 1.2	−23.5 ± 0.8	−17.3 ± 0.6	−14.1 ± 0.5
Size (Exp 3: Group 1) **	0.48 ± 0.05	0.35 ± 0.05	0.32 ± 0.05	0.32 ± 0.05	0.28 ± 0.05
Final position (Exp 3: Group 1) * (mm)	−62.3 ± 2.8	−46.5 ± 2.8	−40.3 ± 3.0	−35.8 ± 3.2	−31.3 ± 2.9

Note 1: The initial sizes (grey background) were the geometrically correct size for each simulated target. Note 2: The initial positions (grey background) are the average of the positions where targets were rendered in the 2D scene for each simulated distance. These were different for each participant due to the different screen sizes. Note 3: The values that are stated after each mean size/positions (±) are the standard errors. * The positions are how far below the horizon the target was placed in the Size-to-Position task. ** The sizes are in proportion to the phone size entered.

## Data Availability

Code for running the experiment, and all the data and analysis scripts, are available at: https://osf.io/khwsd/ (accessed on 28 March 2022).

## Appendix A

**Table A1:** Exp 1—repeated-measures ANOVA—comparing target sizes.

Cases	Sum of Squares	df	Mean Square	F	*p*	η^2^
Simulated Distance	1.796 ^a^	4 ^a^	0.449 ^a^	130.388 ^a^	< 0.001 ^a^	0.124
Residuals	1.116	324	0.003			
Order (initial vs. final)	2.997	1	2.997	33.034	< 0.001	0.207
Residuals	7.348	81	0.091			
Intended Distance ∗ Order	0.096 ^a^	4 ^a^	0.024 ^a^	6.958 ^a^	< 0.001 ^a^	0.007
Residuals	1.116	324	0.003			

Note. Type III Sum of Squares. ^a^ Mauchly’s test of sphericity indicates that the assumption of sphericity is violated (*p* < 0.05).

**Table A2:** Exp 1—repeated-measures ANOVA—comparing target positions.

Cases	Sum of Squares	df	Mean Square	F	*p*	η^2^
Simulated Distance	68699.343 ^a^	4 ^a^	17174.836 ^a^	143.443 ^a^	< 0.001 ^a^	0.639
Residuals	38793.319	324	119.732			

Note. Type III Sum of Squares. ^a^ Mauchly’s test of sphericity indicates that the assumption of sphericity is violated (*p* < 0.05).

**Table A3:** Exp 2—repeated-measures ANOVA—comparing target positions.

Cases	Sum of Squares	df	Mean Square	F	*p*	η^2^
Simulated Distance	149698.623 ^a^	4 ^a^	37424.656 ^a^	385.818 ^a^	< 0.001 ^a^	0.664
Residuals	19788.166	204	97.001			
Order (initial vs. final)	2279.815	1	2279.815	6.582	0.013	0.010
Residuals	17665.312	51	346.379			
Intended Distance ∗ Order	17324.548 ^a^	4 ^a^	4331.137 ^a^	46.905 ^a^	< 0.001 ^a^	0.077
Residuals	18837.248	204	92.339			

Note. Type III Sum of Squares. ^a^ Mauchly’s test of sphericity indicates that the assumption of sphericity is violated (*p* < 0.05).

**Table A4:** Exp 2—repeated-measures ANOVA—comparing target sizes.

Cases	Sum of Squares	df	Mean Square	F	*p*	η^2^
Intended Distance	2.390 ^a^	4 ^a^	0.597 ^a^	68.352 ^a^	< 0.001 ^a^	0.573
Residuals	1.783	204	0.009			

Note. Type III Sum of Squares. ^a^ Mauchly’s test of sphericity indicates that the assumption of sphericity is violated (*p* < 0.05).

**Table A5:** Exp 3: Group 1—repeated-measures ANOVA—comparing target positions.

Cases	Sum of Squares	df	Mean Square	F	*p*	η^2^
Order (initial vs. final)	9757.387	1	9757.387	29.855	< 0.001	0.054
Residuals	13399.824	41	326.825			
Simulated Distance	118578.018 ^a^	4 ^a^	29644.505 ^a^	422.301 ^a^	< 0.001 ^a^	0.658
Residuals	11512.402	164	70.198			
Intended Distance ∗ Order	16233.517 ^a^	4 ^a^	4058.379 ^a^	61.639 ^a^	< 0.001 ^a^	0.090
Residuals	11512.402	164	70.198			

Note. Type III Sum of Squares. ^a^ Mauchly’s test of sphericity indicates that the assumption of sphericity is violated (*p* < 0.05).

**Table A6:** Exp 3: Group 1—repeated-measures ANOVA—comparing target sizes.

Cases	Sum of Squares	df	Mean Square	F	*p*	η^2^
Simulated Distance	0.976 ^a^	4 ^a^	0.244 ^a^	40.487 ^a^	< 0.001 ^a^	0.497
Residuals	0.988	164	0.006			

Note. Type III Sum of Squares. ^a^ Mauchly’s test of sphericity indicates that the assumption of sphericity is violated (*p* < 0.05).

**Table A7:** Exp 3: Group 2—repeated-measures ANOVA—comparing target sizes.

Cases	Sum of Squares	df	Mean Square	F	*p*	η^2^
Order (initial vs. final)	2.018	1	2.018	12.123	0.001	0.193
Residuals	6.826	41	0.166			
Simulated Distance	0.696 ^a^	4 ^a^	0.174 ^a^	64.329 ^a^	< 0.001 ^a^	0.067
Residuals	0.444	164	0.003			
Simulated Distance ∗ Order	0.010 ^a^	4 ^a^	0.002 ^a^	0.916 ^a^	0.456 ^a^	9.502 ×10^−4^
Residuals	0.444	164	0.003			

Note. Type III Sum of Squares. ^a^ Mauchly’s test of sphericity indicates that the assumption of sphericity is violated (*p* < 0.05).

**Table A8:** Exp 3: Group 2—repeated-measures ANOVA—comparing target positions.

Cases	Sum of Squares	df	Mean Square	F	*p*	η^2^
Simulated Distance	46643.277 ^a^	4 ^a^	11660.819 ^a^	77.179 ^a^	< 0.001 ^a^	0.653
Residuals	24778.311	164	151.087			

Note. Type III Sum of Squares. ^a^ Mauchly’s test of sphericity indicates that the assumption of sphericity is violated (*p* < 0.05).
